# *In situ* immobilisation of toxic metals in soil using Maifan stone and illite/smectite clay

**DOI:** 10.1038/s41598-018-22901-w

**Published:** 2018-03-15

**Authors:** Jieyong Ou, Hong Li, Zengguang Yan, Youya Zhou, Liping Bai, Chaoyan Zhang, Xuedong Wang, Guikui Chen

**Affiliations:** 1 Key Laboratory of Agro-Environment in the Tropics, Ministry of Agriculture, South China Agricultural University, Guangzhou, China; 2 State Key Laboratory of Environmental Criteria and Risk Assessment, Chinese Research Academy of Environmental Sciences, Beijing, China; 3 The Key Lab of Resource Environment and GIS, College of Resource Environment and Tourism, Capital Normal University, Beijing, China

## Abstract

Clay minerals have been proposed as amendments for remediating metal-contaminated soils owing to their abundant reserves, high performance, simplicity of use and low cost. Two novel clay minerals, Maifan stone and illite/smectite clay, were examined in the *in situ* immobilisation of soil metals. The application of 0.5% Maifan stone or illite/smectite clay to field soils significantly decreased the fractions of diethylenetriaminepentaacetic acid (DTPA)-extractable Cd, Ni, Cr, Zn, Cu and Pb. Furthermore, reductions of 35.4% and 7.0% in the DTPA-extractable fraction of Cd were obtained with the Maifan stone and illite/smectite clay treatments, respectively, which also significantly reduced the uptake of Cd, Ni, Cr, Zn, Cu and Pb in the edible parts of *Brassica rapa* subspecies *pekinensis*, *Brassica campestris* and *Spinacia oleracea*. Quantitatively, the Maifan stone treatment reduced the metal uptake in *B. rapa* ssp. *Pekinensis*, *B. campestris* and *S*. oleracea from 11.6% to 62.2%, 4.6% to 41.8% and 11.3% to 58.2%, respectively, whereas illite/smectite clay produced reductions of 8.5% to 62.8% and 4.2% to 37.6% in the metal uptake in *B. rapa* ssp. *Pekinensis* and *B. campestris*, respectively. Therefore, both Maifan stone and illite/smectite clay are promising amendments for contaminated soil remediation.

## Introduction

The environmental pollution of soils with heavy metals has become severe due to the rapid growth of industrialisation and urbanisation in the last century^1^. A variety of anthropogenic sources, including metal ore mining and smelting, industrial discharge, automobile emissions and agricultural practices (e.g., the use of synthetic pesticides, industrial waste, and industrial or domestic sludge), have resulted in heavy metal contamination of urban and agricultural soils. In China, the major sources of metals in agricultural soils include atmospheric deposition, livestock manure, fertilisers and agrochemicals, sewage irrigation and sewage sludge^2^, among which atmospheric deposition and livestock manure are the most predominant. Atmospheric deposition is responsible for 43–85% of the total As, Cr, Hg, Ni and Pb input, while livestock manure accounts for approximately 55%, 69% and 51% of the total Cd, Cu and Zn input, respectively. The long-term application of chicken manure to the vegetable soils of Jinhua city, Zhejiang province of China has resulted in the significant accumulation of Pb, Cd, Cu, and Cr in the soils^3^. In particular, 80% of the arable land in the surveyed area was contaminated with Cd as a result of the long-term application of chicken manure. In fact, the heavy metal contamination of soils is a wide-spread environmental problem in China. According to the report from the National Soil Pollution Survey carried out from 2005 to 2013, 19.4% of the arable land survey sites failed to attain the Soil Environment Quality Standards (SEQS), with dominant contaminants of Cd, Cr, Ni, Zn, Cu and Pb. The concentrations of these contaminants were above the SEQS in 7.0%, 1.1%, 4.8%, 0.9%, 2.1% and 1.5%, respectively, of the arable land survey sites^4^. The contamination of arable soils by heavy metals can strongly impact the food quality and safety and subsequently pose a risk to human health through the food chain^5,6^.

To address the problem of heavy metal contamination in soils, an array of remediation technologies, including physical remediation, chemical remediation and biological remediation, have been developed. Historically, chemical fixation had been widely used to solidify/stabilise heavy metals in industrially contaminated soils. However, only in recent years has this technique been frequently used to immobilise metals in arable soils. In chemical fixation, reagents or amendments are added into the contaminated soil to react with heavy metals and form insoluble or nearly immobile, minimally toxic materials. The formation of these materials decreases the migration of heavy metals to water, plants and other environmental media, which is interpreted as the successful remediation of the soils^7^. Compared to other treatment technologies, the chemical fixation of metals in arable soils bears several benefits: first, it reduces the mobility and bioavailability of soil metals and subsequently alleviates the impacts of metals on plants and groundwater; second, it has little impact on normal crop production in contaminated soils; and third, it saves time and does not require that the cultivating patterns of crops be changed.

Numerous amendments have been proposed and tested in the immobilisation of metals in soil. The most widely applied amendments include lime, limestone, clay minerals, zeolites, phosphates, organic composts and carbon nanotubes^8–15^. More recently, various clay minerals, including sepiolite, illite, calcite, goethite, montmorillonite, bentonite, and kaolinite, have been extensively examined^16^. Unfortunately, most of these studies were limited to laboratory-scale incubation or pot trials^17–19^, and only a few field studies demonstrated the effectiveness of clay minerals in the immobilisation of soil metals^20,21^. Therefore, more efforts are needed to develop novel clay minerals for the *in situ* immobilisation of soil metals in the field. In the present study, two novel clay minerals, Maifan stone and illite/smectite clay, were applied to soils to determine the effectiveness of the minerals in immobilising soil metals. The bioaccumulation of Cd, Cr, Ni, Zn, Cu and Pb in three foliar vegetable crops, *Brassica rapa* spp. *pekinensis*, *Brassica campestris* and *Spinacia oleracea*, were used to evaluate the efficiency of the amendments in alleviating the phytotoxicity of heavy metals in the crops. The results may assist us in developing new clay minerals for the remediation of metal-contaminated soils.

## Results

### Effects of the amendments on the extractability of metals in soil

The effects of the amendments on the mobility and bioavailability of metals in soil were evaluated by extracting the metals using diethylenetriaminepentaacetic acid (DTPA) As expected, the addition of Maifan stone and illite/smectite clay to soils resulted in a reduction in the fractions of extractable Cd, Ni, Cr, Zn, Cu and Pb (Table 1). The addition of Maifan stone to the soils of the plots planted with *B rapa* ssp. *pekinensis* resulted in a reduction of the DTPA-extractable fractions of Cd, Ni, Cr, Zn, Cu and Pb by 18.0%, 31.3%, 25.1%, 26.0%, 22.3% and 22.3%, respectively, while the addition of illite/smectite clay to the soils of the plots planted with *B. rapa* ssp. *pekinensis* resulted in a reduction of the DTPA-extractable fractions of Cd, Ni, Cr, Zn, Cu and Pb by 7.0%, 26.7%, 32.2%, 17.0%, 19.4% and 17.7%, respectively (Table 1). In the plots planted with *B. campestris*, the addition of Maifan stone to the soils reduced the fractions of DTPA-extractable Cd, Ni, Cr, Zn, Cu and Pb, by 9.6%, 22.9%, 36.3%, 8.2%, 8.3% and 5.7%, respectively, and the addition of illite/smectite clay reduced the fractions of DTPA-extractable Cd, Ni, Cr, Zn, Cu and Pb, by 5.1%, 38.7%, 39.3%, 5.6%, 12.2% and 14.2%, respectively. Additionally, the addition of Maifan stone to the soils of the plots planted with *S. oleracea* resulted in a reduction of the DTPA-extractable fractions of Cd, Ni, Cr, Zn, Cu and Pb by 35.4%, 37.2%, 8.5%, 21.1%, 25.0% and 21.0%, respectively (Table 1).

**Table 1:** DTPA-extracted metal concentrations (mg/kg) in soils planted with *Brassica rapa* spp. *pekinensis*, *Brassica campestris*, and *Spinacia oleracea*^a^.

Metal	*B rapa ssp. pekinensis*			*B. campestris*			*S. oleracea*	
	Untreated	Maifan stone	Illite/smectite	Untreated	Maifan stone	Illite/smectite	Untreated	Maifan stone
Cd	0.12 ± 0.01a	0.10 ± 0.01b	0.11 ± 0.01ab	0.11 ± 0.01a	0.10 ± 0.01a	0.11 ± 0.01a	0.12 ± 0.01a	0.08 ± 0.01b
Cr	0.33 ± 0.05a	0.25 ± 0.02b	0.22 ± 0.01b	0.35 ± 0.02a	0.23 ± 0.01b	0.21 ± 0.01b	0.19 ± 0.01a	0.17 ± 0.004b
Ni	1.35 ± 0.21a	0.91 ± 0.11b	0.98 ± 0.14b	1.30 ± 0.16a	1.00 ± 0.06b	0.78 ± 0.10c	0.62 ± 0.15a	0.40 ± 0.13a
Pb	1.70 ± 0.17a	1.32 ± 0.33a	1.4 ± 0.24a	1.94 ± 0.09a	1.83 ± 0.09a	1.65 ± 0.10b	1.84 ± 0.13a	1.45 ± 0.32a
Cu	5.65 ± 0.78a	4.42 ± 0.59b	4.56 ± 0.18b	5.83 ± 0.36a	5.34 ± 0.31b	5.15 ± 0.13b	5.19 ± 0.76a	3.89 ± 0.68b
Zn	10.32 ± 1.20a	7.66 ± 1.24b	8.58 ± 1.11ab	8.93 ± 1.17a	8.20 ± 1.12a	8.40 ± 0.19a	9.08 ± 1.44a	7.18 ± 1.13a

^a^Means ± standard deviations followed by different letters are significantly different (*p* < 0.05).

The total content of each metal in the various soil samples generally remained at a similar level and showed no significant difference (*p* < 0.05) among the plots planted with the same vegetable (Table 2). Nonetheless, the addition of Maifan stone and illite/smectite clay slightly but significantly changed the total soil contents of Cr, Ni and Zn in the soils of the plots planted with *B. rapa* ssp. *pekinensis*, *B. campestris* and *S. oleracea*. In the plots planted with *B. rapa* ssp. *pekinensis*, the addition of Maifan stone and illite/smectite clay resulted in 6.2% and 5.7% increases, respectively, of the total Cr in the soil relative to that in the plots without amendments. However, in the plots planted with *B. campestris*, the addition of illite/smectite clay resulted in 9.1% and 11.1% reductions of the total soil Ni and Zn, respectively. In the plots planted with *S. oleracea*, the addition of Maifan stone resulted in a 7.7% reduction in the total soil Ni.

**Table 2:** Soil pH values and total metal concentrations (mg/kg) in soils planted with *Brassica rapa* spp. *pekinensis*, *Brassica campestris*, and *Spinacia oleracea*^*a*^.

pH/Metal	*B rapa ssp. pekinensis*			*B. campestris*			*S. oleracea*	
	Untreated	Maifan stone	Illite/smectite	Untreated	Maifan stone	Illite/smectite	Untreated	Maifan stone
pH	8.09 ± 0.30a	8.02 ± 0.10a	8.12 ± 0.27a	7.91 ± 0.40a	7.48 ± 0.03a	7.82 ± 0.48a	8.25 ± 0.21a	7.49 ± 0.40b
Cd	0.61 ± 0.03a	0.62 ± 0.03a	0.61 ± 0.03a	0.60 ± 0.03a	0.61 ± 0.02a	0.58 ± 0.01a	0.55 ± 0.03a	0.55 ± 0.01a
Cr	40.41 ± 1.03b	42.63 ± 1.21a	42.72 ± 0.96a	40.72 ± 3.32ab	42.07 ± 0.97a	37.13 ± 2.91b	31.15 ± 1.66a	31.14 ± 1.04a
Ni	14.97 ± 0.57a	15.54 ± 0.41a	15.18 ± 0.40a	15.14 ± 0.74a	15.37 ± 0.42a	13.92 ± 0.76b	12.31 ± 0.45a	11.49 ± 0.32b
Pb	9.31 ± 0.30a	9.32 ± 1.13a	9.28 ± 0.43a	9.51 ± 0.49a	9.36 ± 0.24a	9.10 ± 0.73a	7.19 ± 0.62a	7.19 ± 0.45a
Cu	18.72 ± 1.25a	19.36 ± 0.72a	18.33 ± 0.79a	18.80 ± 1.38a	19.61 ± 0.54a	18.14 ± 0.56a	15.94 ± 1.44a	16.83 ± 0.99a
Zn	70.66 ± 6.26a	74.35 ± 4.82a	70.51 ± 3.00a	71.57 ± 4.88a	73.11 ± 3.63a	63.59 ± 4.75b	59.04 ± 0.23a	62.20 ± 5.62a

^a^Means ± standard deviations followed by different letters are significantly different (*p* < 0.05).

### Effects of the amendments on the uptake of metals in vegetables

**Figure 1:**
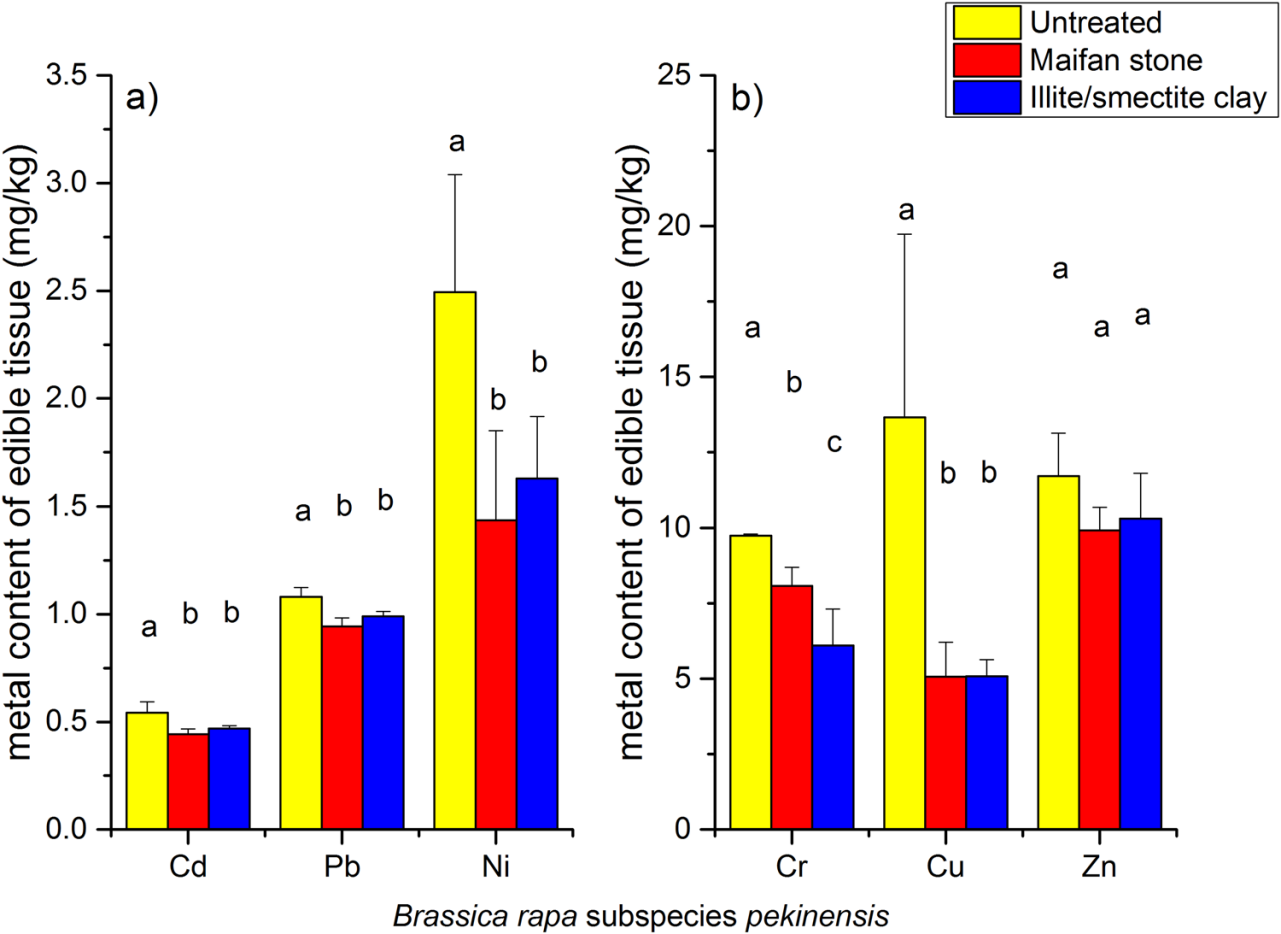
Effects of Maifan stone and illite/smectite clay on the concentrations of toxic metals in the edible parts of *Brassica rapa* spp. *pekinensis*. Error bars represent standard deviations, and the means with different letters are significantly different from each other (*p* < 0.05).

**Figure 2:**
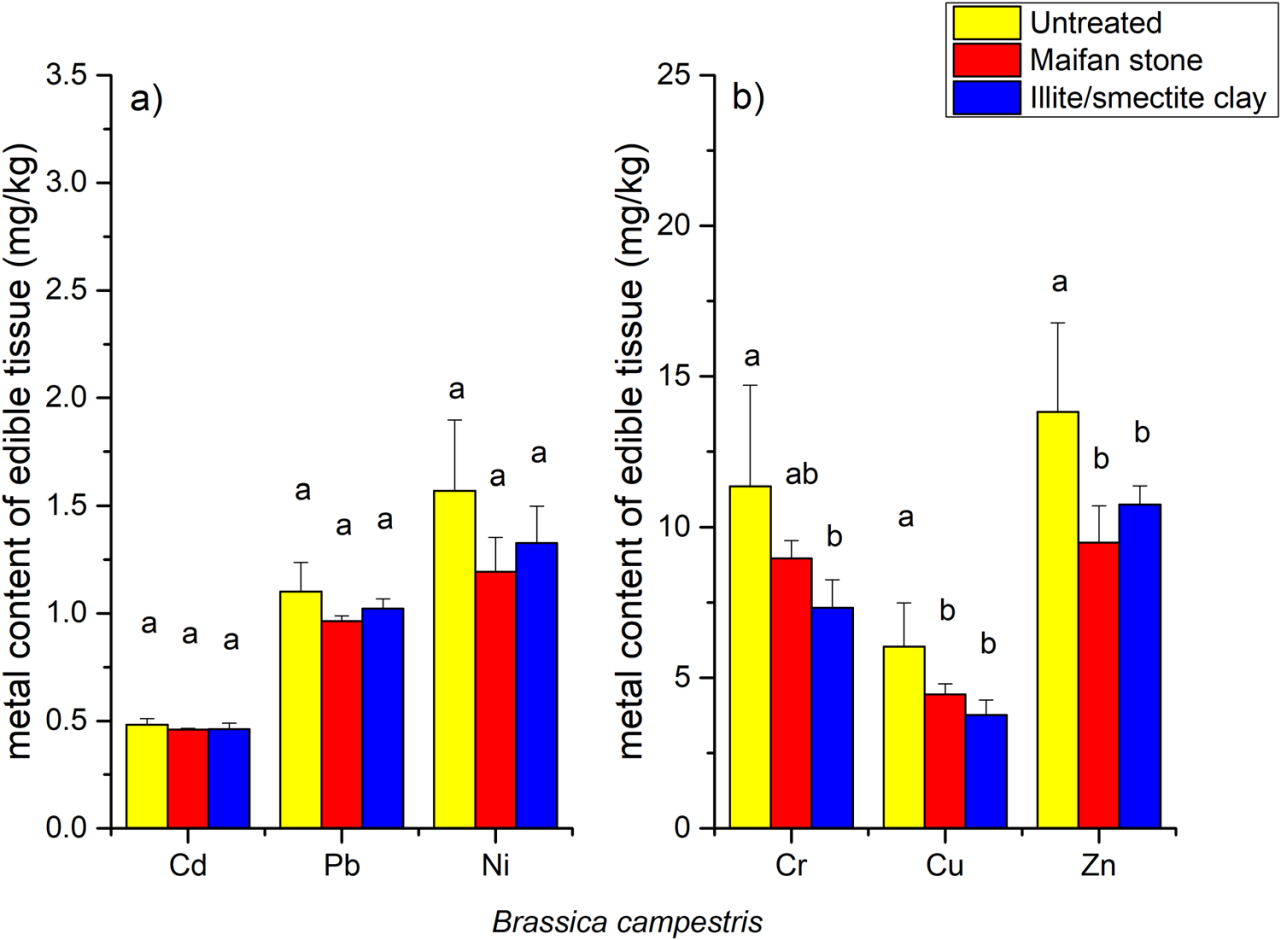
Effects of Maifan stone and illite/smectite clay on the concentrations of toxic metals in the edible parts of *Brassica campestris*. Error bars represent standard deviations, and the means with different letters are significantly different from each other (*p* < 0.05).

**Figure 3:**
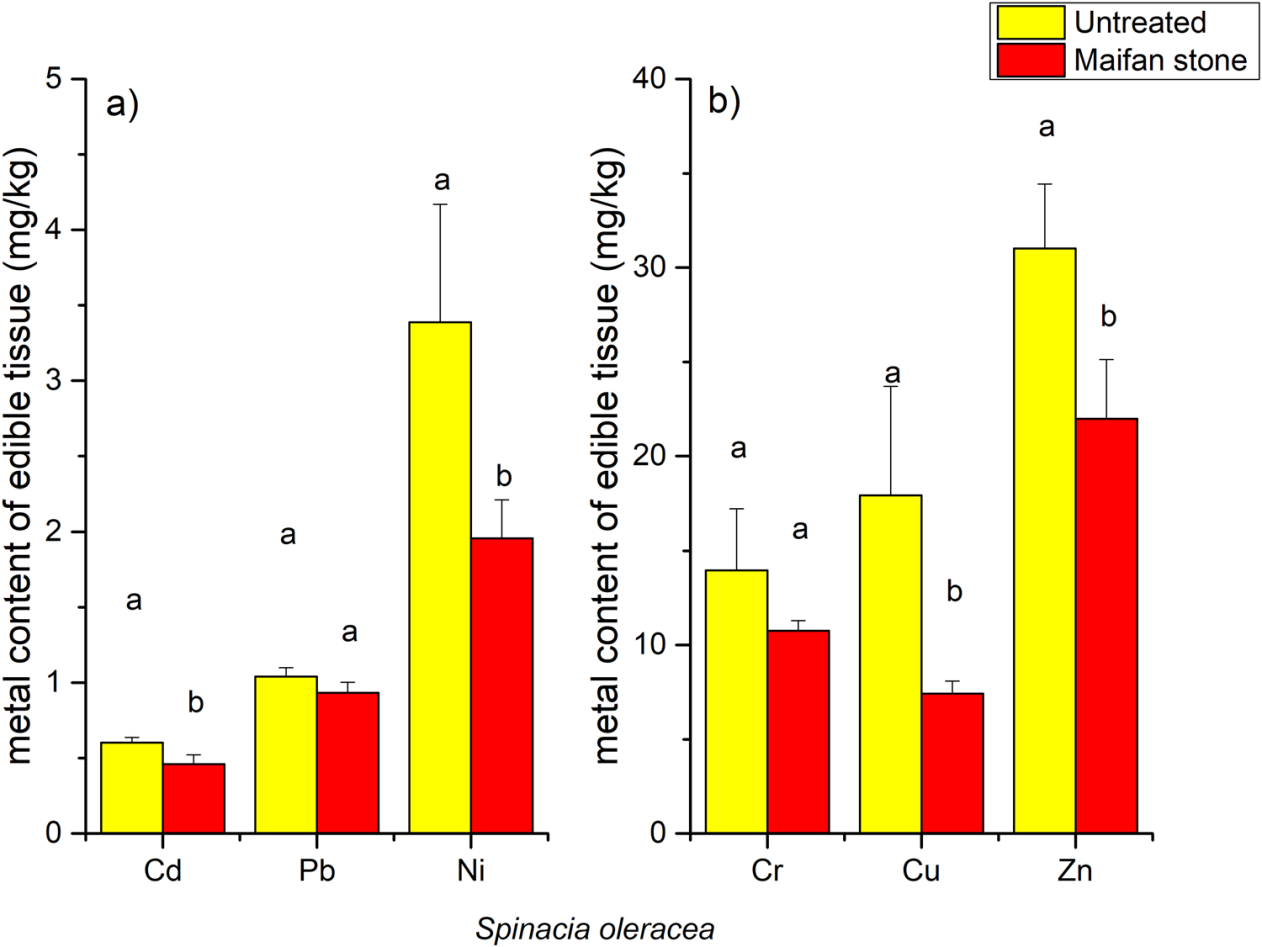
Effects of Maifan stone on the concentrations of toxic metals in the edible parts of *Spinacia oleracea*. Error bars represent standard deviations, and the means with different letters are significantly different from each other (*p* < 0.05).

The addition of 0.5% Maifan stone or illite/smectite clay to the soils generally reduced the uptake of metals in the edible parts of the three vegetables. The application of Maifan stone in the soils resulted in a 11.6% to 62.2% reduction in the Cd, Ni, Cr, Zn, Cu and Pb contents in *B. rapa* ssp. *pekinensis* (Fig. 1), and the application of illite/smectite clay resulted in an 8.5% to 62.8% reduction in the Cd, Ni, Cr, Zn, Cu and Pb contents (Fig. 1). For both Maifan stone and illite/smectite clay, the strongest effect was on Cu, while the weakest effect was on Pb. Maifan stone and illite/smectite clay also reduced the uptake of metals in the edible parts of *B. campestris*, although to a lesser extent (Fig. 2). The total reductions in the uptake of Cd, Ni, Cr, Zn, Cu and Pb were 4.6%, 41.8%, 21.0%, 31.4%, 26.1% and 12.4%, respectively, after treatment with the Maifan stone amendment. The total reductions in the uptake of Cd, Ni, Cr, Zn, Cu and Pb were 4.2%, 35.3%, 35.5%, 22.2%, 37.6% and 7.2%, respectively, after treatment with the illite/smectite clay amendment. Treatment of the soils with a 0.5% Maifan stone amendment also resulted in 25.1%, 43.9%, 23.7%, 32.7%, 58.2% and 11.3% reductions in the Cd, Ni, Cr, Zn, Cu and Pb contents, respectively, in the edible parts of *S. oleracea* (Fig. 3).

The effects of the amendments on the bio-concentration factors (BCFs) of Cd, Ni, Cr, Zn, Cu and Pb in the three vegetables were also evaluated. Compared to the corresponding controls, the treatments with Maifan stone and illite/smectite clay amendments reduced the BCFs of Cd, Ni, Cr, Zn, Cu and Pb in all three vegetables (Supplementary Fig. S4), indicating that Maifan stone and illite/smectite clay were effective soil amendments for reducing the accumulation of metals in the vegetables.

### Effects of the amendments on plant growth and the soil pH

The effects of the amendments on plant growth were also investigated to determine if the amendment itself exerted adverse effects on plant growth. Compared to the controls, the treatment of the soils with Maifan stone and illite/smectite clay amendments had no significant impact on the plant biomass (Table 3).

**Table 3:** Biomass (g/plant in dry weight) of *Brassica rapa* spp. *pekinensis*, *Brassica campestris*, and *Spinacia oleracea*^a^.

Treatment	*B rapa ssp. pekinensis*	*B. campestris*	*S. oleracea*
Untreated	12.61 ± 3.72a	12.80 ± 3.50a	8.36 ± 3.74a
Maifan stone	14.75 ± 3.52a	11.02 ± 3.67a	9.25 ± 3.21a
Illite/smectite	13.59 ± 2.94a	12.27 ± 3.38a	—

^a^Mean values ± standard deviations (*n = *15) with the same letter in the same column indicate no significant difference (*p < *0.05).

The soils used in the experiments were calcareous soils with pH values ranging from 7.48 to 8.25. The treatment of the soils with Maifan stone and illite/smectite clay amendments had no significant impact on the soil pH (Table 2).

## Discussion

A range of common clay minerals, such as natural sepiolite, palygorskite, and bentonite, have been widely examined for potential application in the remediation of contaminated arable soils^8^. Clay minerals are generally able to decrease the fractions of extractable/bioavailable heavy metals in the soil. For example, Hodson *et al*. used 0.01 M CaCl_2_ and DTPA to evaluate the ability of bone meal (finely ground, poorly crystalline apatite) to immobilise metals in soil and found that this material could reduce the availability of soil metals through the formation of metal phosphates^22^. Zhang *et al*. evaluated the efficiency of phosphate rock in remediating Cd- and Pb-contaminated soils and found that a 40 g/kg loading of phosphate rock could decrease the available soil Cd fraction by 83.09%, while an 80 g/kg loading of phosphate rock could decrease the available soil Pb fraction by 23.79%^23^. Lv *et al*. examined the efficiency of sodium bentonite, bentonite, zeolite, and diatomite in remediating Cd–contaminated soils and found that application ratios of 20, 30, 50 and 40 g/kg, respectively, of these four minerals could reduce the available soil Cd fraction by 21.40%, 27.63%, 27.24% and 32.30%, respectively^24^. Sun *et al*. carried out both pot and field experiments to determine the efficiency of sepiolite in stabilising soil Cd and found that the application of 1% to 5% sepiolite could reduce the toxicity characteristic leaching procedure (TCLP)-extractable fraction of Cd from 0.6% to 49.6% and from 4.0% to 32.5% in the pot and field experiments, respectively^25^. In the present study, we conducted field experiments to evaluate the potential of two clay minerals, Maifan stone and illite/smectite clay, for use in the *in situ* immobilisation of toxic metals in soil. The application of 0.5% Maifan stone to field soils planted with *B. rapa* ssp. *pekinensis*, *B. campestris* and *S. oleracea* was found to reduce the DTPA-extractable fraction of Cd in soils by 9.6% to 35.4% (Table 1). Additionally, the application of 0.5% illite/smectite clay to soils planted with *B. rapa* ssp. *pekinensis* and *B. campestris* reduced the DTPA-extractable fraction of Cd by 5.1% and 7.0%, respectively. Similarly, the application of Maifan stone and illite/smectite clay reduced the DTPA-extractable fractions of Ni, Cr, Zn, Cu and Pb in soils planted with *B. rapa* ssp. *pekinensis*, *B. campestris* or *S. oleracea*. These findings implied that Maifan stone and illite/smectite clay have potential for application as amendments in the remediation of metal-contaminated soils. Note that the application rate (0.5%) of Maifan stone and illite/smectite clay in the present study was lower than that in other studies, in which 0.5% to 10% amendment loadings were often used for the *in situ* immobilisation of soil metals^26^. Therefore, the immobilisation efficiencies of Maifan stone and illite/smectite clay in the present study were generally lower than those of other clay minerals in other studies, as could be expected based on the loading. The immobilisation efficiencies of Maifan stone, illite/smectite clay and the clay minerals reported in other studies are shown in Supplementary Table S1.

The concentrations of metals in leafy and root vegetables are normally correlated with the fraction of phytoavailable metals^10^. Thus, the changes in the metal extractability in response to the amendment treatments were expected to directly affect metal uptake in vegetables. As summarised by Zhang *et al*.^27^, clay minerals such as sepiolite, palygorskite and bentonite successfully reduced the metal contents in the edible parts of vegetables. The introduction of 1% to 5% sepiolite into Cd-contaminated soils in the field reduced Cd uptake by 22.8% to 61.4% in *S. oleracea*^23^. In the present study, the treatment of Cd-contaminated soils with 0.5% Maifan stone resulted in 18.0%, 9.6%, and 35.4% reductions in the uptake of Cd in *B. rapa* ssp. *pekinensis*, *B. campestris* and *S. oleracea*, respectively (Figs 1–3). Additionally, the treatment of Cd-contaminated soils with 0.5% illite/smectite clay resulted in 7.0% and 5.1% reductions in the uptake of Cd in *B. rapa* ssp. *pekinensis* and *B. campestris*, respectively. Reductions in the uptake of Ni, Cr, Zn, Cu and Pb in *B. rapa* ssp. *pekinensis*, *B. campestris* or *S. oleracea* were also observed after the treatment of the soils with Maifan stone or illite/smectite clay. Changes in the accumulation of metals in vegetables are also clearly reflected by shifts in the BCFs. The treatment of the soils with Maifan stone or illite/smectite clay reduced the BCFs of Cd, Ni, Cr, Zn, Cu and Pb in the three vegetables (Supplementary Fig. S4). Taken together, these results confirmed the findings that Maifan stone and illite/smectite clay could reduce metal bioavailability in soil and implies again that the two clay minerals have potential for application in the remediation of metal-contaminated soils.

Due to genotypic variations, different crop species exhibit different metal accumulation abilities^28^. For instance, Alexander *et al*. examined six vegetables that are commonly grown in metal-contaminated soils and found that regardless of the extent of metal accumulation, the order of metal uptake was lettuce > spinach > carrot > onion > pea > bean^19^. In the present study, *B. rapa* ssp. *pekinensis* and *B. campestris* exhibited similar accumulation abilities for Cd, Ni, Cr, Zn, Cu and Pb (Figs 1 and 2). However, *S. oleracea* seemed to accumulate more Ni, Cr, Cu and Zn than *B. rapa* ssp. *pekinensis* and *B. campestris* despite the similar accumulations of Cd and Pb in these three vegetables. The ultimate aim in the remediation of metal-contaminated arable soils is to ensure food safety and reduce the potential risk to human health. Common minerals, such as sepiolite, palygorskite, and bentonite, have been successfully used to reduce the heavy metal contents in the edible parts of rice and vegetables^27^. However, the final contents differed due to differences in the soil pollution status, soil properties, plant growth, and applied amendment dose. In the present study, when the dry-weight-based levels of Cd, Ni, Cr, Zn, Cu and Pb in the edible parts of the vegetables were converted to fresh-weight-based levels (Table 4) using the measured moisture contents of each plant tissue, the levels of Cd, Ni, Zn, Cu and Pb in *B. rapa* ssp. *pekinensis*, *B. campestris* and *S. oleracea* were all under the maximum allowable limits established by the National Food Hygiene Standard of China (NFHSC, GB 2762-2012). However, the fresh-weight-based levels of Cr in the edible parts of *B. campestris* and *S. oleracea* exceeded the maximum allowable limit (0.5 mg/kg) of the NFHSC (Table 4). The application of 0.5% Maifan stone or illite/smectite clay to the soils reduced the Cr content in *B. campestris* to levels below 0.5 mg/kg, indicating that the use of Maifan stone or illite/smectite clay could ensure food safety in the production of *B. campestris*. However, even though the application of 0.5% Maifan stone also significantly reduced the uptake of Cr in *S. oleracea*, the fresh-weight-based levels of Cr in the edible parts of *S. oleracea* remained above the maximum allowable limit of the NFHSC. Nevertheless, even though the Cr concentration in *S. oleracea* grown on soils treated with 0.5% Maifan stone was not reduced to below the specific standard limit, the significant reduction in the Cr content achieved in the edible parts might contribute to the protection of the consumer’s health by minimising Cr intake.

**Table 4:** Metal concentrations (mg/kg, fresh weight) in vegetables grown in soils treated with Maifan stone and illite/smectite clay^a^.

Metal	*B rapa ssp. pekinensis*			*B. campestris*			*S. oleracea*		MPL^b^
	Untreated	Maifan stone	Illite/smectite	Untreated	Maifan stone	Illite/smectite	Untreated	Maifan stone	
Cd	0.023 ± 0.007	0.016 ± 0.001	0.019 ± 0.001	0.025 ± 0.002	0.021 ± 0.001	0.024 ± 0.002	0.044 ± 0.006	0.031 ± 0.005	0.2
Cr	0.402 ± 0.087	0.300 ± 0.044	0.245 ± 0.061	0.580 ± 0.185	0.412 ± 0.060	0.386 ± 0.049	1.007 ± 0.218	0.721 ± 0.074	0.5
Pb	0.045 ± 0.011	0.035 ± 0.002	0.040 ± 0.003	0.056 ± 0.009	0.044 ± 0.004	0.054 ± 0.005	0.076 ± 0.010	0.062 ± 0.006	0.3

^a^Mean values ± standard deviations.

^b^MPL = Maximum permissible level established by the NFHSC (GB 2762-2012).

Numerous mechanisms have been proposed to explain the decrease in the metal availability in amended soils. Cation exchange, adsorption, surface complexation and precipitation are considered the primary mechanisms responsible for metal immobilisation by amendments^8,17^. For clay minerals, the remediation mechanisms mainly include liming, precipitation, and sorption^16^. The increase in the soil pH that results from the addition of sepiolite into Cd–contaminated soils was proposed to be the most important factor responsible for immobilising Cd and reducing its uptake in spinach^29^. This hypothesis was confirmed in another study, in which sepiolite was found to increase the pH of acidic paddy soils and decrease the phytoavailable fractions of heavy metals in the soil^18^. In fact, most clay minerals, including sepiolite, palygorskite, and bentonite, are alkaline minerals. The addition of these clay minerals into soils usually results in an increase in the soil pH and, consequently, has a positive effect on the remediation of metal-contaminated acidic soils. Therefore, unexpectedly, most studies involving the use of clay minerals for soil remediation were conducted in acidic soils, and only a few were conducted in neutral and alkaline soils^27^. However, this trend does not mean that clay minerals are ineffective in the remediation of neutral or alkaline soils. For example, bentonite has been shown to reduce the exchangeable fractions of Cd and Pb in alkaline soils by 11.1% to 42.5% and 20.3% to 49.3%, respectively^30^. Maifan stone and illite/smectite clay are also alkaline minerals. The application of 0.5% Maifan stone or illite/smectite clay to the calcareous Beijing fluvo-aquic soil (pH 7.48-8.25) in the present study had no significant impact on the soil pH (Table 2). However, the phytoavailability of Cd, Ni, Cr, Zn, Cu and Pb in the soil and their uptake in *B. rapa* ssp. *pekinensis*, *B. campestris* and *S. oleracea* were significantly reduced. As no pH effect was evident, chemical/physical fixation/adsorption by Maifan stone and illite/smectite clay may play a dominant role in decreasing the metal mobility. Outer-sphere complex formation, inner-sphere complex formation, lattice diffusion, and isomorphic substitution within the mineral lattice have been proposed as possible mechanisms for heavy metal ion sorption at the mineral/water interface^31,32^. The large number of pores on the surface and in the interior portions of the particles of Maifan stone and illite/smectite clay (Supplementary Fig. S2) may introduce new sorption sites that may immobilise heavy metals in soils through specific adsorption processes.

In summary, the potential of two clay minerals, Maifan stone and illite/smectite clay, for use in the *in situ* immobilisation of toxic metals in soil was evaluated. The application of Maifan stone or illite/smectite clay to field soils significantly reduced the DTPA-extractable fractions of Cd, Ni, Cr, Zn, Cu and Pb. Additionally, the contents of Cd, Ni, Cr, Zn, Cu and Pb in the edible parts of *B. rapa* ssp. *pekinensis*, *B. campestris* and *S. oleracea* decreased significantly as a result of the application of Maifan stone or illite/smectite clay to the soils. Moreover, the field experiments also showed that both Maifan stone or illite/smectite clay had no effect on the soil pH or the vegetables biomass. These findings highlight the potential use of these two clay minerals in the remediation of metal-contaminated arable soils. Future studies should focus on the immobilisation processes and influencing factors to obtain better insight into the immobilisation mechanisms of Maifan stone and illite/smectite clay.

## Materials and Methods

### Materials

Maifan stone and illite/smectite clay were used as amendments for the *in situ* immobilisation of metals in the field. Maifan stone was purchased from Qingmao S & T Limited, Hebei, China, and illite/smectite clay was purchased from Zhongkenada S&T Limited, Beijing, China. Dust powders of the two clay minerals with particle sizes < 100 μm were used in the experiments (Supplementary Fig. S1). Scanning electron microscopy (SEM) analysis confirmed that Maifan stone had a high degree of crystalline cuboids, while illite/smectite contained crystalline scales (Supplementary Fig. S2). A large number of pores were present on the surface and interior portions of the particles of both Maifan stone and illite/smectite clay. The elemental composition of Maifan stone clay (energy dispersive spectroscopy (EDS) analysis) consisted of a mixture of O, Mg, Al, Si, K, Fe, Na, and S, and the composition of illite/smectite clay (EDS analysis) was a mixture of O, Mg, Al, Si, K, Fe, Ca, and Ti (Supplementary Text S1 and S2).

### Field experiments

Field experiments for the immobilisation of heavy metals (Cd, Cr, Ni, Zn, Cu and Pb) by Maifan stone and illite/smectite clay were carried out on farmland located in the Daxing District of Beijing, China (Supplementary Fig. S4). The land had been used to grow vegetables for more than thirty years, and metals had accumulated in the soil due to the long-term application of organic fertilisers (chicken manure). The soil used in the field experiments had a pH of 7.5, an organic matter content of 39.53 g/kg, and a cation-exchange capacity (CEC) of 12.71 cmol/kg. The baseline concentrations of Pb, Cd, Cr, Cu, Zn and Ni in the experimental soils were 7.08, 0.54, 31.05, 15.94, 59.04 and 11.47 mg/kg, respectively. The Cd concentration in the experimental soil was approximately two-fold higher that the standard level (0.3 mg/kg) of the Chinese SEQS.

The field experiments were conducted in a block split-spot design with 4 replications of each control (no added amendment) and amendment treatment. Each replicate of the control or treatment was artificially arranged to reduce the effects of spatial heterogeneity. The size of each plot was 4 m^2^ (2 m × 2 m). Before addition of the amendment, 4 kg of organic fertiliser (compost chicken manure) was applied to the soil of each plot according to local farming practices. One week after the addition of fertilisers, the amendments were applied at a ratio of 0.5% (W/W) to the surface of each plot before being ploughed into the soil to a depth of 20 cm. After this procedure, the soils were equilibrated for one week, and then the seeds of *B. rapa* ssp. *pekinensis*, *B. campestris* and *S. oleracea* were sown into the Maifan stone-treated soils, while only the seeds of *B. rapa* ssp. *pekinensis* and *B. campestris* were sown into the illite/smectite clay-treated soils. A sufficient number of seeds were sown to guarantee healthy germination, and then the seedlings were thinned to 60 plants per plot.

After 40 days of growth, 5 subsamples of the vegetables in each plot were collected and combined for chemical analysis. The fresh vegetable samples were placed in clean plastic bags and transported to the laboratory for sample treatment. The vegetables were divided into roots and edible parts, rinsed with tap water followed by deionised water, and then oven-dried at 65 °C for 48 h to constant weight, and the dry weights (DW) were recorded.

After harvesting the vegetables, 5 subsamples of the soils (0–20 cm) were evenly collected from each plot and then composited to give a single representative sample for each plot soil. The collected soils were air-dried, ground to pass through a 0.149 mm (100 mesh) nylon sieve and then used for chemical analysis.

### Chemical analysis

The contents of six heavy metals (Cd, Cr, Ni, Zn, Cu and Pb) in the edible parts of each vegetable species were determined. Briefly, the oven-dried plant samples were ground, and then 0.5 g of the sample was transferred to a Teflon tube (7 ml) containing 6 ml of a mixture of H_2_O_2_ and HNO_3_ (1:2, v/v) and digested for 48 h at 130 °C after standing overnight. The concentrations of Cd, Cr, Ni, Zn, Cu and Pb were measured using inductively coupled plasma mass spectrometry (ICP-MS) (Agilent 7500c, Agilent Technologies, CA, USA). Blank and tea materials (GBW10052) (National Information Infrastructure for CRMs, China) were included for quality assurance, and the recoveries of the six metals were all within the certified limits.

Both the total and the extractable metal contents in the soil were determined. The total concentrations of Cd, Cr, Ni, Zn, Cu and Pb in the soil were determined according to the Chinese standard method HJ 803-2016. Briefly, 0.1 g of the soil sample was digested in 6 ml of a mixture of HCl and HNO_3_ (3:1, v/v) in a Teflon tube (7 ml) for 130 min at 185 °C. The resultant solution was filtered, transferred to a flask (100 ml) and then used for measurement of the metal concentrations. The total concentrations of Cd, Cr, Ni, Zn, Cu and Pb were measured using ICP-MS (Agilent 7500c, Agilent Technologies, CA, USA). Blanks and reference soil materials (GBW07410) (National Information Infrastructure for CRMs, Beijing, China) were included for quality assurance, and the recoveries of the metals from the soil materials were all within the certified limits.

The measurement of the extractable metal fractions in the soil was performed according to Chinese standard method HJ 804-2016. A DTPA solution containing 0.1 mol/L triethanolamine, 0.01 mol/L CaCl_2_, and 0.005 mol/L diethylene triamine pentaacetic acid (DTPA) was used for the extraction of the soil metals. In brief, 10.0 g of the soil sample was treated with 20 ml of the DTPA solution (pH 7.3) and shaken for 2 h at 20 °C. The resultant suspension was centrifuged at 3000 g for 10 min, and the supernatant was collected and filtered through 0.45 μm membranes. The extractable Cd, Cr, Ni, Zn, Cu and Pb concentrations were measured by ICP-MS (Agilent 7500c, Agilent Technologies, CA, USA).

### Statistical analysis

The results were presented as arithmetic means with standard deviations. The BCF was calculated using Eq. (1).

1 $${\rm{BCF}}=\frac{{\rm{metal}}\,{\rm{content}}\,{\rm{in}}\,{\rm{plant}}\,({\rm{DW}})}{{\rm{Metal}}\,{\rm{content}}\,{\rm{in}}\,{\rm{soil}}\,({\rm{DW}})}$$

Duncan’s multiple-range test after analysis of variance (ANOVA) was used to determine significant differences (*p* < 0.05) among the treatments in *B. rapa* ssp. *pekinensis* and *B. campestris*. A Student t-test was performed to evaluate the difference (*p* < 0.05) among the treatments in *S. oleracea*. Statistical analysis was done through the SPSS statistical program version 22.0.

### Data availability

All data generated or analysed during this study are included in this published article (and its Supplementary Information files).

## Electronic supplementary material

Supplementary Information
