# Supplementary material for: In situ immobilisation of toxic metals in soil using Maifan stone and illite/smectite clay
Source: Sci Rep. 2018 Mar 15;8:4618. doi: 10.1038/s41598-018-22901-w (PMC5854592; doi:10.1038/s41598-018-22901-w)
Supplement: Supplementary file 1 — Supplementary Information [file 41598_2018_22901_MOESM1_ESM.doc]

**SUPPLEMENTARY INFORMATION**

In situ immobilisation of toxic metals in soil using Maifan stone and illite/smectite clay

Jieyong Ou^1,2^, Hong Li^2,3^, Zengguang Yan^2^*, Youya Zhou^2^, Liping Bai^2^, Chaoyan Zhang^2^, Xuedong Wang^3^, Guikui Chen^1^*

^1^ Key Laboratory of Agro-Environment in the Tropics, Ministry of Agriculture, P.R. China, South China Agricultural University, Guangzhou, China

^2^ State Key Laboratory of Environmental Criteria and Risk Assessment, Chinese Research Academy of Environmental Sciences, Beijing, China

^3^ The Key Lab of Resource Environment and GIS, College of Resource Environment and Tourism, Capital Normal University, Beijing, China

* Corresponding author

E-mail: [yanzg@craes.org.cn](mailto:yanzg@craes.org.cn) (ZGY); [guikuichen@scau.edu.cn](mailto:guikuichen@scau.edu.cn) (GKC)

**Contents**

**Supplementary Figure S1:** The powders of Maifan stone and illite/smectite clay

**Supplementary Figure S2:** SEM image of Maifan stone and illite/smectite clay

**Supplementary Figure S3:** Field experiments in the greenhouse

**Supplementary Figure S4:** BCF values of Cd, Ni, Cr, Zn, Cu and Pb in *Brassica rapa subspecies pekinensis*, *Brassica campestris* and *Spinacia oleracea*

**Supplementary Text S1:** Elemental composition of Maifan stone analyzed by Energy Dispersive X-ray Spectroscopy (EDS)

**Supplementary Text S2:** Elemental composition of illite/smectite clay analyzed by Energy Dispersive X-ray Spectroscopy (EDS)

**Supplementary Table S1:** Clay minerals used as amendments for remediation of heavy metal-polluted soils

**Supplementary Figure S1:
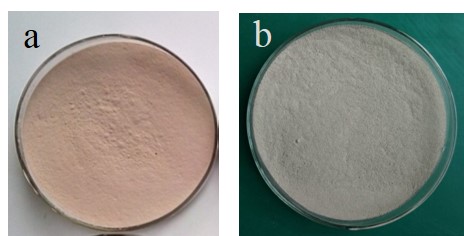
**

**Supplementary Figure S1: The powders of illite/smectite clay(a) and Maifan stone(b)**

Maifan stone and illite/smectite clay were used as amendments for in-situ immobilization of metals in field. Maifan stone was purchased from Qingmao S & T Limited, Hebei, and illite/smectite clay was from Zhongkenada S&T Limited, Beijing, China. Dust powder of the two clay minerals with particles size < 100 μm were used in the experiments


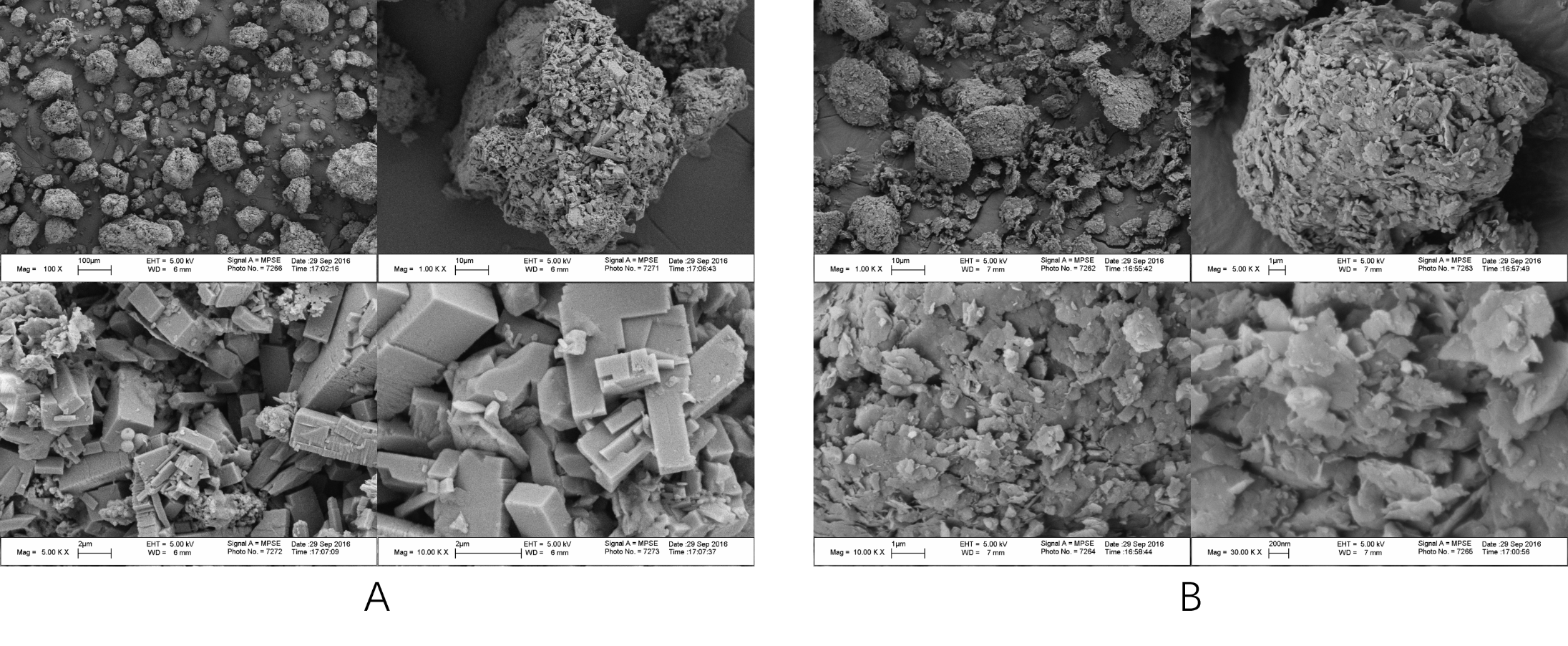
**Supplementary Figure S2:**

**Supplementary Figure S2: SEM** **image of Maifan stone (A) and illite/smectite clay(B)**


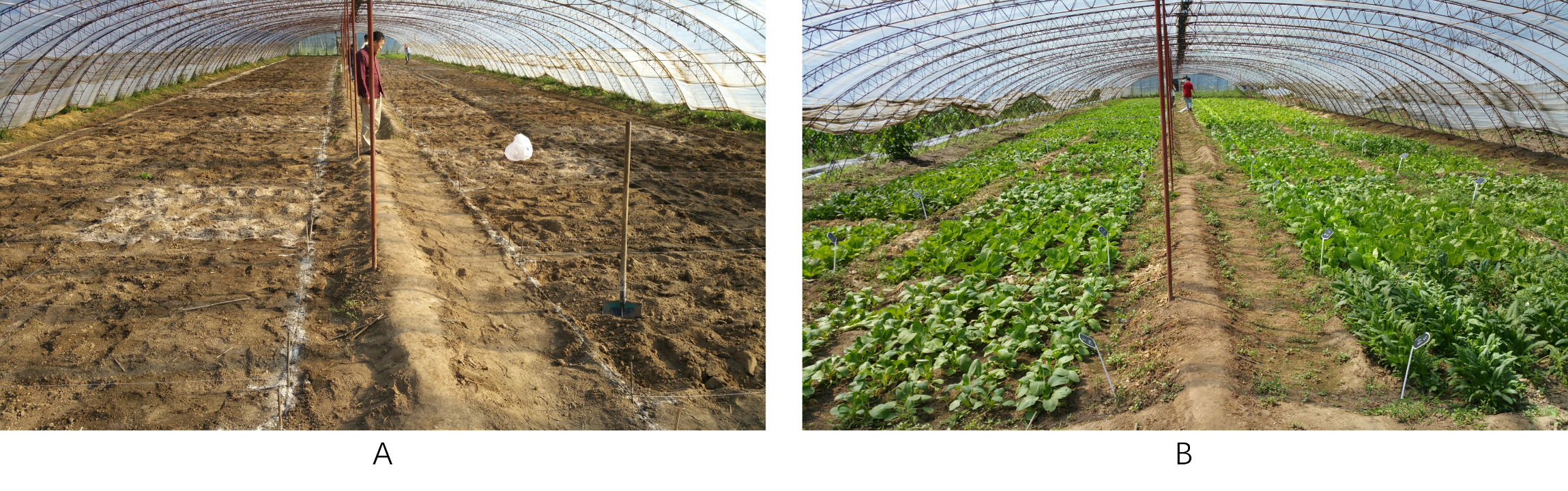
**Supplementary Figure S3:**

**Supplementary Figure S3: Field experiments in the greenhouse**

1. The field experiments were a block split-spot design with 4 replications for the control and each amendment treatment. Each replicate of the control or the same treatment was artificially arranged to reduce the effects of spatial heterogeneity. The size of each plot was 4 m^2^ (2 m × 2 m). Before amendment addition, 4 kg of organic fertilizers (compost chicken manure) was applied to the soils of each plot according to local farming practices. One week after the addition of fertilizers, the amendments were applied at a rate of 0.5% (W/W) to the surface of each plot before being ploughed into the soil to a depth of 20 cm. After this, the soils were equilibrated for one week and then the seeds of *B. rapa ssp. pekinensis*, *B. campestris* and *S. oleracea* were sown into the Maifan stone treated soils while only the seeds of *B. rapa ssp. pekinensis* and *B. campestris* were sown into the illite/smectite clay treated soils. Sufficient seeds were sown to guarantee healthy germination and then seedlings were thinned to 60 plants per plot.
2. after 40 days of growth, 5 subsamples of vegetables in each plot were collected and combined for chemical analysis. The fresh vegetable samples were put in clean plastic bags and transported to laboratory for sample treatment. The vegetables were splited into roots and edible parts, and then rinsed with tap water followed by deionized water and then oven dried at 65°C for 48 h to constant weight and dry weights (DW) were recorded.


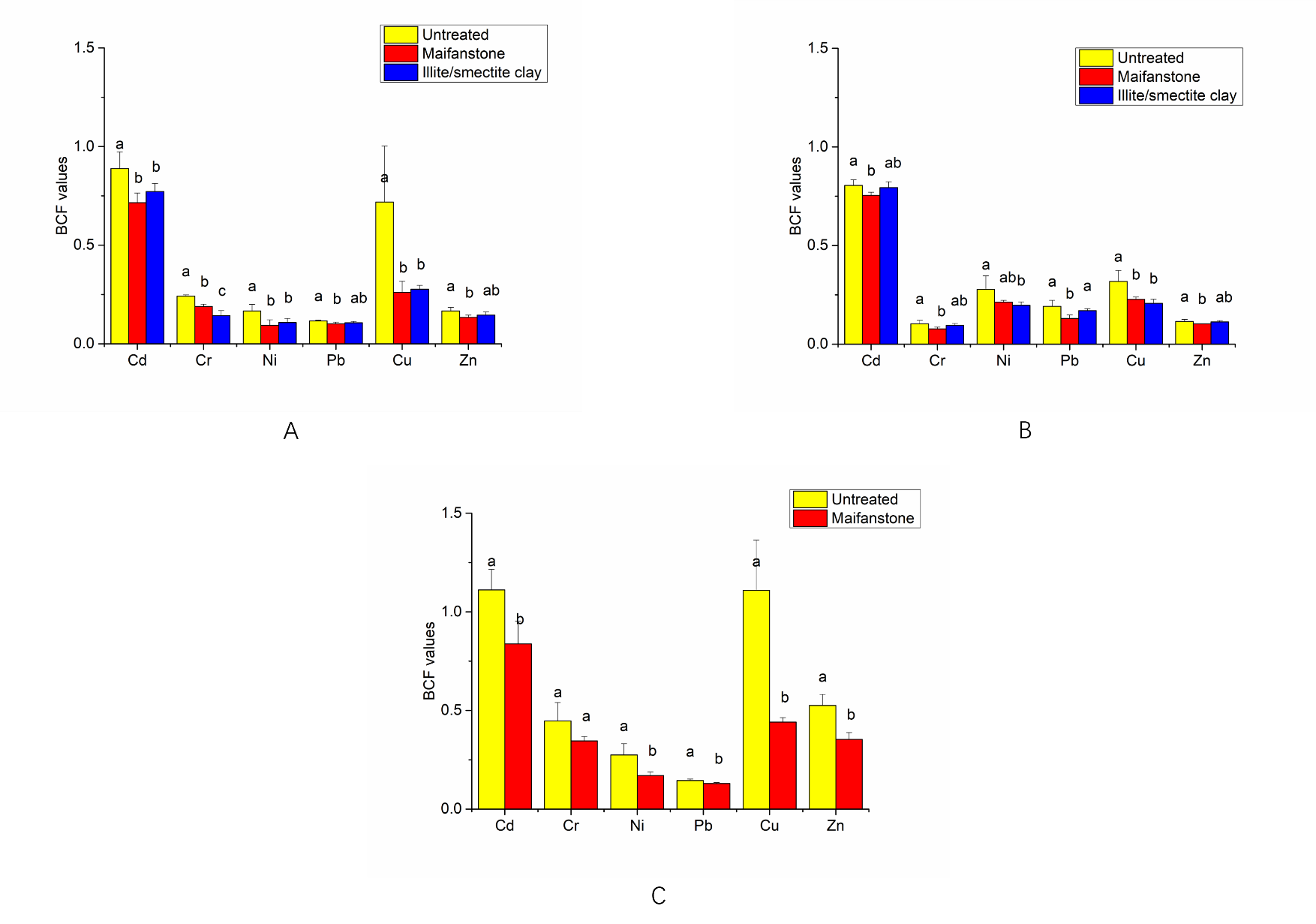
**Supplementary Figure S4:**

**Supplementary Figure S4: BCF values of Cd, Ni, Cr, Zn, Cu and Pb in *Brassica rapa subspecies pekinensis*(A), *Brassica campestris*(B) and *Spinacia oleracea*(C),**


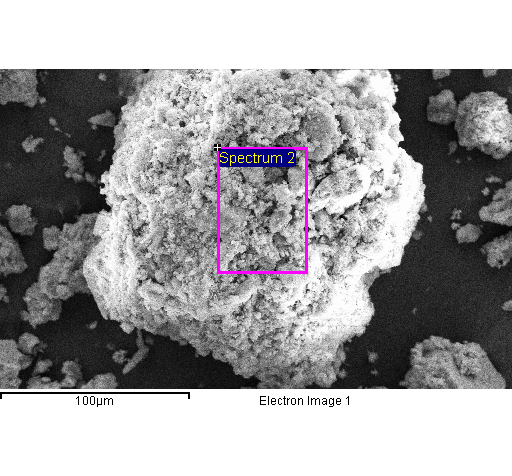

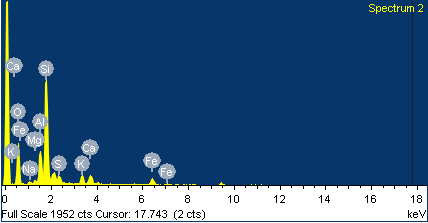

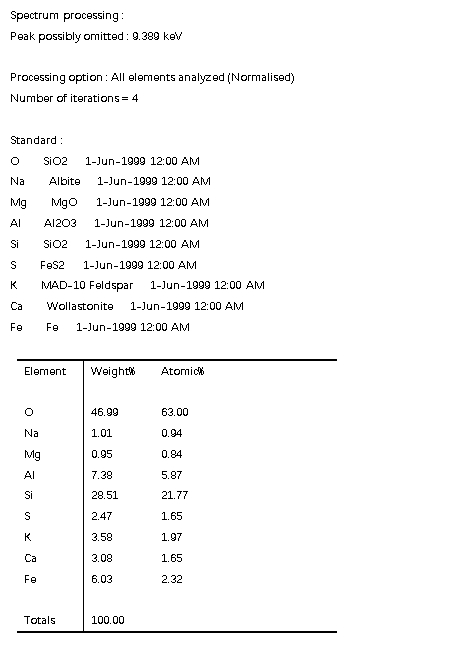
**Supplementary Text S1:**


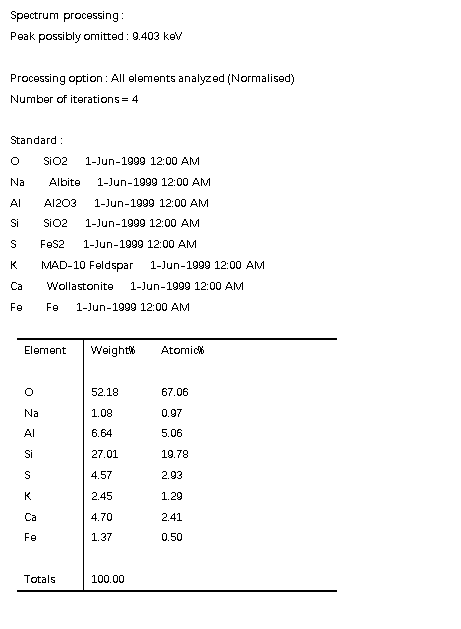

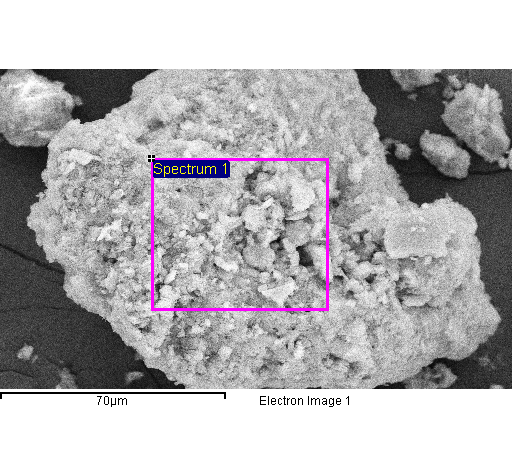

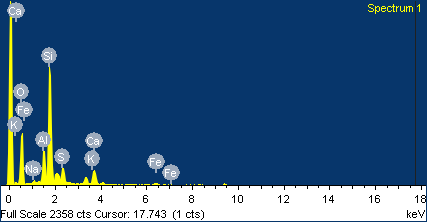
**Supplementary Text S1: Elemental composition of Maifan stone analyzed by Energy Dispersive X-ray Spectroscopy (EDS)**


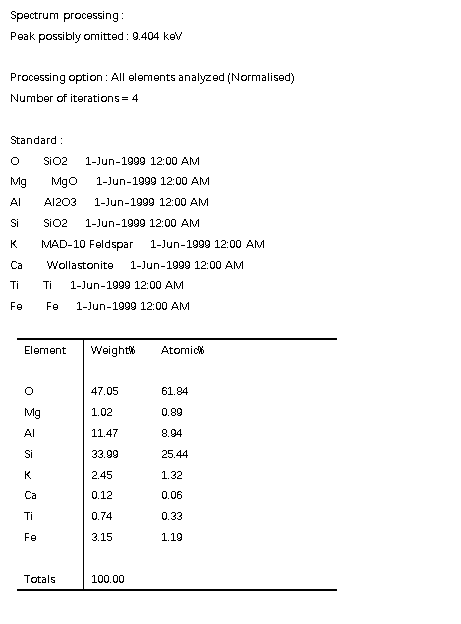

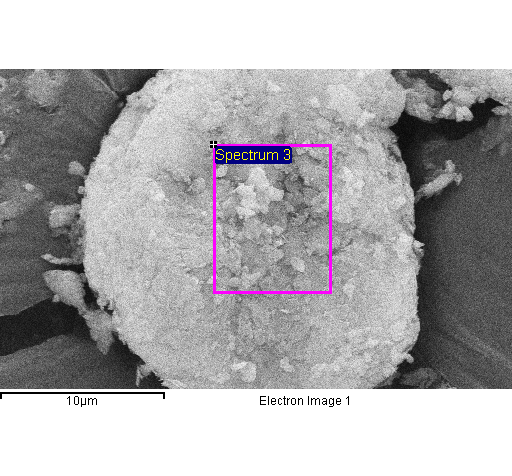

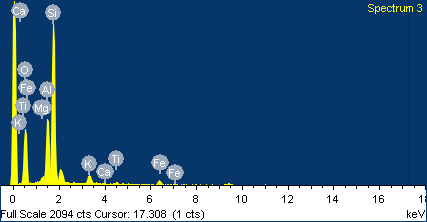
**Supplementary Text S2:**

**
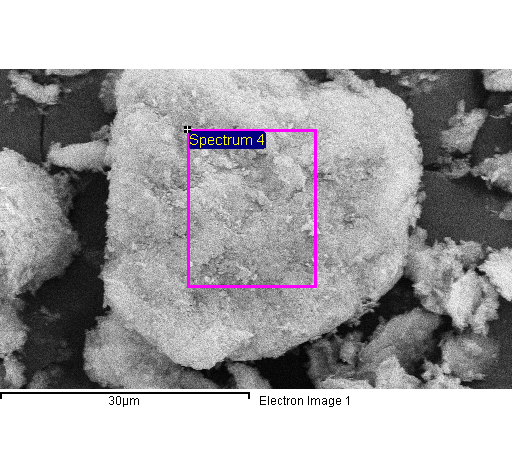

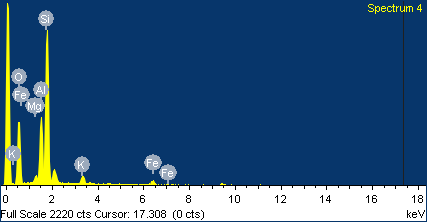
**

**Supplementary Text S2: Elemental composition of illite/smectite clay analyzed by Energy Dispersive X-ray Spectroscopy (EDS**
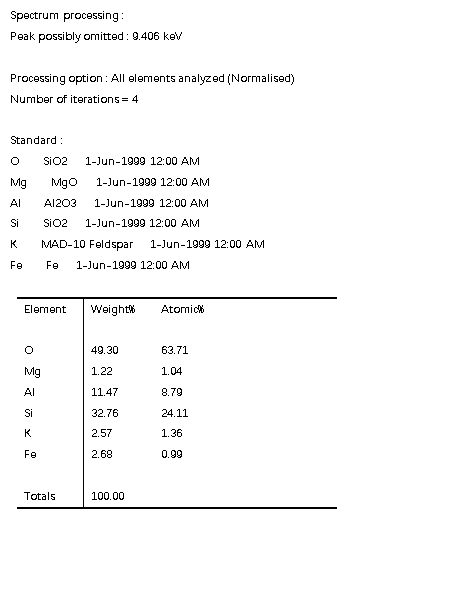
**)**

**Supplementary Table S1: Clay minerals used as amendments for remediation of heavy metal-polluted soils**

| Study | Amendment(s) | Metal(s) | Soil pH | Amendment dosage | Plant | Scale^a^ | Amendment effect on the uptake of heavy metals by plants | Amendment effect on the bioavailability of heavy metals in soil^b^ |
| --- | --- | --- | --- | --- | --- | --- | --- | --- |
| The present study | Maifan stone | Cd, Ni, Cr, Zn, Cu, Pb | 7.48-8.25 | 0.50% | *Brassica rapa* subspecies *pekinensis*, *Brassica campestris*, *Spinacia oleracea* | F | Reduced Cd, Ni, Cr, Zn, Cu and Pb contents in three vegetables (by 25.1%, 43.9%, 23.7%, 32.7%, 62.2% and 12.4%, in maximum, respectively) | Decreased concentrations of DTPA-extractable Cd, Ni, Cr, Zn, Cu and Pb (by 35.4%, 37.2%, 36.3%, 26.0%, 25.0% and 22.3%, in maximum, respectively) |
|  | Illite/smectite clay |  |  |  | *Brassica rapa* subspecies *pekinensis*, *Brassica campestris* |  | Reduced Cd, Ni, Cr, Zn, Cu and Pb contents in two vegetables (by 13.7%, 35.3%, 37.4%, 22.2%, 62.8% and 8.5%, in maximum, respectively) | Decreased concentrations of DTPA-extractable Cd, Ni, Cr, Zn, Cu and Pb (by 7.0%, 38.7%, 39.3%, 17.0%, 19.4% and 17.7%, in maximum, respectively) |
| Hodson *et al.* (2000)^1^ | Bonemeal | Zn, Pb, Cd, Ni, Cu | 2.7-7.1 | 2% |  | I |  | Significantly reduced CaCl2- and DTPA- extractable Zn, Pb, Cd, Ni, Cu (by 12.5%-18.7% and 40.2%-47.9%, respectively) with an increase in the pH of soils |
| Zhang *et al.* (2009)^2^ | Phosphate rock | Cd, Zn, Cu, Pb | 7.0-8.1 | 4%, 8%, 12% |  | I |  | Decreased the available Cd, Zn, Cu and Pb with increasing dosages (by 83.1%, 37.2%, 31.2% and 23.8%, in maximum, respectively) |
|  | Furfural dreg |  |  |  |  |  |  |  |
|  | Weathered coal |  |  |  |  |  |  |  |
| Lv *et al.* (2009)^3^ | Na-modified bentonite | Cd | 8.1 | 2%, 3%, 4%, 5% | *Brassica napus L.* | F | Decreased Cd contents in plants by 3.1%-15.6% with increasing dosages | Reduced HCl-extractable Cd by 21.4%-31.3% with increasing dosages |
|  | Bentonite |  |  |  |  |  |  |  |
|  | Zeolite |  |  |  |  |  |  |  |
|  | Diatomite |  |  |  |  |  |  |  |
| Sun *et al.* (2016)^4^ | Sepiolite | Cd | 4.48, 6.19, 7.76 | 1%, 5% | Spinach | P, F | Significantly reduced plant Cd uptake in the pot and field experiments (by 14.4%-84.1% and 22.8%-61.4%, respectively) | Significantly reduced TCLP-extractable Cd in the pot and field experiments (by 0.6%-49.6% and 4.0%-32.5%, respectively) |
| Zhang *et al.* (2011)^5^ | Montmorillonite | Cu | 3.99 | 1%, 2%, 4%, 8% |  | I |  | Decreased acid exchangeable Cu by 24.7% in maximum |
| Liang *et al.* (2014)^6^ | Sepiolite | Cd | 5.5 | 0.75, 1.50, 2.25 kg/m^2^ | Rice | F | Decreased Cd concentrations in brown rice by 52.3%-73.6% and 40.2%-47.9% for two cultivars | Significantly decreased all extractable Cd fractions |
| Sun *et al.* (2015)^7^ | Bentonite | Cd, Pb | 8.2 | 0.5%, 1%, 3%, 5% | Rice | P | reduced rice Cd and Pb uptake by 44.3% and 7.8% respectively | Increased residual fractions of Cd and Pb (54.3% and 10.0%, respectively), Reduced exchangeable fractions of Cd and Pb (42.5% and 49.3%, respectively) |

^a^ I = incubation; F = field; P= pot

^b^ TCLP = toxicity characteristic leaching procedure; DTPA = diethylenetriaminepentaacetic acid

**References**

1. Hodson ME, Valsami-JonesÉ. Bonemeal additions as a remediation treatment for metal contaminated soil. *Environ. Sci. Technol.* **34**,3501-3507, doi: 10.1021/es990972a (2000).
2. Zhang LJ, Zhang Y, Liu DH. Remediation of soils contaminated by heavy metals with different amelioration materials. *Soils*. **41**, 420-424, doi: 10.13758/j.cnki.tr.2009.03.005 (2009).
3. Lv L. *et al.* Study on remediation of the soil contaminatd with cadmium by applying four minerals. *Journal of Agriculture University of Hebei*, **32**. 1-5(2009). In Chinese.
4. Sun, Y. *et al.* Reliability and stability of immobilization remediation of Cd polluted soils using sepiolite under pot and field trials. *Environ Pollut* **208**, 739-746, doi:10.1016/j.envpol.2015.10.054 (2016).
5. Zhang, G., Lin, Y. & Wang, M. Remediation of copper polluted red soils with clay materials. *Journal of Environmental Sciences* **23**, 461-467, doi:10.1016/s1001-0742(10)60431-7 (2011).
6. Liang, X. *et al.* In situ field-scale remediation of Cd polluted paddy soil using sepiolite and palygorskite. *Geoderma* **235-236**, 9-18, doi:10.1016/j.geoderma.2014.06.029 (2014).
7. Sun, Y., Li, Y., Xu, Y., Liang, X. & Wang, L. In situ stabilization remediation of cadmium (Cd) and lead (Pb) co-contaminated paddy soil using bentonite. *Applied Clay Science* **105-106**, 200-206, doi:10.1016/j.clay.2014.12.031 (2015).
